# Microenvironmental immune cell alterations across the spectrum of nodular lymphocyte predominant Hodgkin lymphoma and T-cell/histiocyte-rich large B-cell lymphoma

**DOI:** 10.3389/fonc.2023.1267604

**Published:** 2023-10-03

**Authors:** Christos Panayi, Ayse U. Akarca, Alan D. Ramsay, Ananth G. Shankar, Brunangelo Falini, Miguel A. Piris, David Linch, Teresa Marafioti

**Affiliations:** ^1^ Department of Cellular Pathology, University College London Hospitals NHS Foundation Trust, London, United Kingdom; ^2^ University College London (UCL) Cancer Institute, University College London, London, United Kingdom; ^3^ Children and Young People’s Cancer Services, University College London Hospitals NHS Foundation Trust, London, United Kingdom; ^4^ Institute of Hematology and Center for Haemato-Oncological Research (CREO), University of Perugia and Santa Maria della Misericordia Hospital, Perugia, Italy; ^5^ Pathology Department, Instituto de Investigación Sanitaria Fundación Jiménez Díaz, Madrid, Spain; ^6^ Research Department of Haematology, Cancer Institute, University College London, London, United Kingdom

**Keywords:** nodular lymphocyte predominant Hodgkin lymphoma (NLPHL), T-cell/histiocyte-rich large B-cell lymphoma, tumor microenvironment, immune checkpoints, lymphoma biology, multispectral immunofluorescence

## Abstract

**Background:**

The clinicopathological spectrum of nodular lymphocyte predominant Hodgkin lymphoma (NLPHL), also known as nodular lymphocyte predominant B-cell lymphoma, partially overlaps with T-cell/histiocyte-rich large B-cell lymphoma (THRLCBL). NLPHL histology may vary in architecture and B-cell/T-cell composition of the tumour microenvironment. However, the immune cell phenotypes accompanying different histological patterns remain poorly characterised.

**Methods:**

We applied a multiplexed immunofluorescence workflow to identify differential expansion/depletion of multiple microenvironmental immune cell phenotypes between cases of NLPHL showing different histological patterns (as described by Fan et al, 2003) and cases of THRLBCL.

**Results:**

FOXP3-expressing T-regulatory cells were conspicuously depleted across all NLPHL cases. As histology progressed to variant Fan patterns C and E of NLPHL and to THRLBCL, there were progressive expansions of cytotoxic granzyme-B-expressing natural killer and CD8-positive T-cells, PD1-expressing CD8-positive T-cells, and CD163-positive macrophages including a PDL1-expressing subset. These occurred in parallel to depletion of NKG2A-expressing natural killer and CD8-positive T-cells.

**Discussion:**

These findings provide new insights on the immunoregulatory mechanisms involved in NLPHL and THLRBCL pathogenesis, and are supportive of an increasingly proposed biological continuum between these two lymphomas. Additionally, the findings may help establish new biomarkers of high-risk disease, which could support a novel therapeutic program of immune checkpoint interruption targeting the PD1:PDL1 and/or NKG2A:HLA-E axes in the management of high-risk NLPHL and THRLBCL.

## Introduction

Nodular lymphocyte predominant Hodgkin lymphoma (NLPHL) is a rare lymphoma with incidence of up to 0.3 cases per 100,000 person years (1, 2). The sparse neoplastic “lymphocyte predominant” (LP) cells of NLPHL retain a germinal centre B-cell programme including expression of markers such as CD20, CD79a and BCL6; ongoing somatic hypermutation; and engagement with PD1-expressing T-follicular helper cells (Tfh) through immunological synapses (3–6). The latter frequently manifest histologically as T-cell rosettes around LP cells. Furthermore, NLPHL and T-cell/histiocyte-rich large B-cell lymphoma (THRLBCL) share overlapping histological, molecular and clinical features (7–10). As these characteristics are in contrast with those of classical Hodgkin lymphoma (cHL), a shift in terminology to “nodular lymphocyte predominant B-cell lymphoma” is now acceptable or preferred in both contemporary classification systems (11, 12).

**Figure 1:**
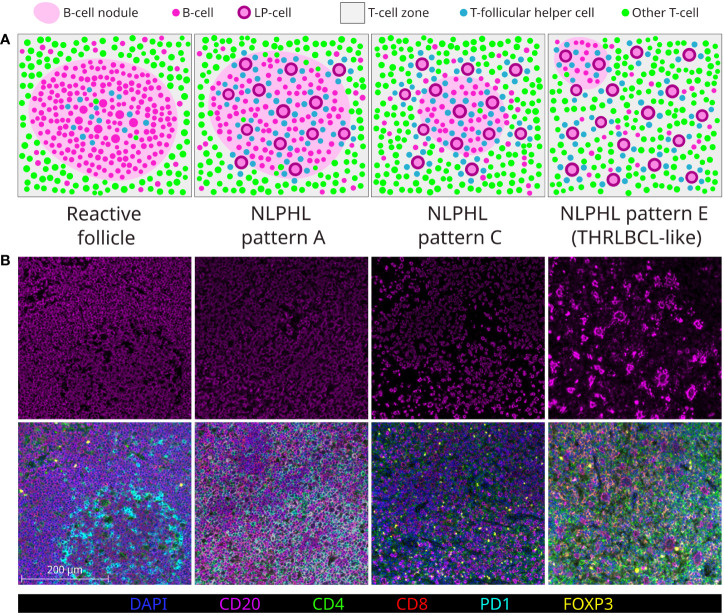
Major NLPHL histological patterns, compared to a reactive follicle. Demonstrated by graphical diagram **(A)**; and example fields from multispectral immunofluorescence multichannel image tiles **(B)**, including component CD20 signal (upper row) and composite of all components signals of the lymphocyte panel (lower row).

Clinical outcomes in NLPHL correlate to the architectural patterns of the tumour microenvironment (TME) in relation to the LP cells, as described by Fan et al. (13). Typical cases show LP cells residing in B-cell rich nodular/serpiginous formations (patterns A/B) and present with localised disease following an indolent course (1, 2, 14). Less typically, LP cells reside in a T-cell rich TME which is either extra-nodular (pattern C), intra-nodular (pattern D), diffuse (THRLBCL-like pattern E), or within “moth eaten” diffuse B-cell areas (pattern F). These variant patterns are associated with clinically advanced and high-risk disease across adolescent and adult populations (15–17). Patterns A, C and E are most widely reported in diagnostic histopathology practice (each illustrated in **Figure 1A**).

In the era of increasingly limited biopsy material, all NLPHL patterns may either be ambiguously mixed or difficult to assign, whilst NLPHL pattern E may mimic lymphocyte-rich cHL (LRcHL) or be indistinguishable from THRLBCL if a coincident nodular focus is not sampled (18). Furthermore, whilst patterns A/B and E are clearly at opposite histological extremes of the NLPHL spectrum, how the intermediate pattern C and less common patterns D/F should be regarded in attempts to histologically grade NLPHL is less clear (19).

In this study, we applied a single-cell resolution multiplexed immunofluorescence and innovative digital image analysis workflow to characterise differential frequency of TME immune cell phenotypes between the major NLPHL patterns and THRLBCL. As well as advancing understanding of the tumour immuno-biology linking these entities, immunological characterisation of TME constituents in this way may yield additional objective methods of stratifying the spectrum of NLPHL and potential biomarkers of high-risk disease.

## Methods

Multiplexed multispectral immunofluorescence was performed on archival formalin-fixed paraffin-embedded cases histologically classified as NLPHL (n=15) or THRLBCL (n=4). For comparison, cases of LRcHL (n=4) and tonsillar follicular hyperplasia control tissue were also included. Histological diagnoses were confirmed by a consultant and trainee histopathologist (TM/CP). NLPHL cases were subcategorised as purely pattern A/B (NLPHL-pA; n=6), pattern C dominant (NLPHL-pC; n=3), and pattern E dominant (NLPHL-pE; n=6) with patterns D/F not being distinctly encountered.

Three panels were applied using the ‘Opal 7-Color’ system (Akoya Biosciences, Marlborough, MA, USA), adapted from previously optimised protocols (20). These targeted distinct immune cells of interest, as follows:

− *Lymphocyte panel*: CD20, CD4, CD8, PD1, FOXP3 and DAPI
− *Cytotoxic/Natural Killer (NK) cell panel*: CD8, CD16, CD56, NKG2A, Granzyme-B (GZMB), Granulysin (GNLY) and DAPI
− *Macrophage panel*: CD68, CD163, CD206, PDL1 and DAPI

Multispectral images were acquired and spectrally unmixed on the Vectra3 platform and companion InForm software (Akoya Biosciences). Representative multi-channel image tiles of equal size (670x501 microns; 1348x1008 pixels) were exported from each case for further analysis (number of tiles analysed shown in **Table 1**). Exemplary fields of composite multi-channel images tiles from each case type are shown in **Figure 1B**.

**Table 1:** Number of analysed cases and images tiles.

Case type	Lymphocyte panel		Cytotoxic/NK panel		Macrophage panel	
	Cases*	Image tiles	Cases*	Image tiles	Cases*	Image tiles
**Tonsil**	-	15	-	55	-	62
**NLPHL-pA**	6 (5 + 1)	160	5 (4 + 1)	285	6 (5 + 1)	174
**NLPHL-pC**	3 (3 + 0)	58	3 (3 + 0)	89	3 (3 + 0)	98
**NLPHL-pE**	6 (3 + 3)	219	6 (3 + 3)	181	6 (3 + 3)	278
**THRLBCL**	4	187	4	95	4	152
**LRcHL**	4	302	4	313	4	288

NLPHL, nodular lymphocyte predominant Hodgkin lymphoma; -pA, pure pattern A; -pC, pattern C dominant; -pE, pattern E dominant; THRLBCL, T-cell/histiocyte-rich large B-cell lymphoma; LRcHL, lymphocyte-rich classical Hodgkin lymphoma.

*Parentheses indicate the contribution of NLPHL cases by clinical service setting (adolescent + adult).

**Figure 2:**
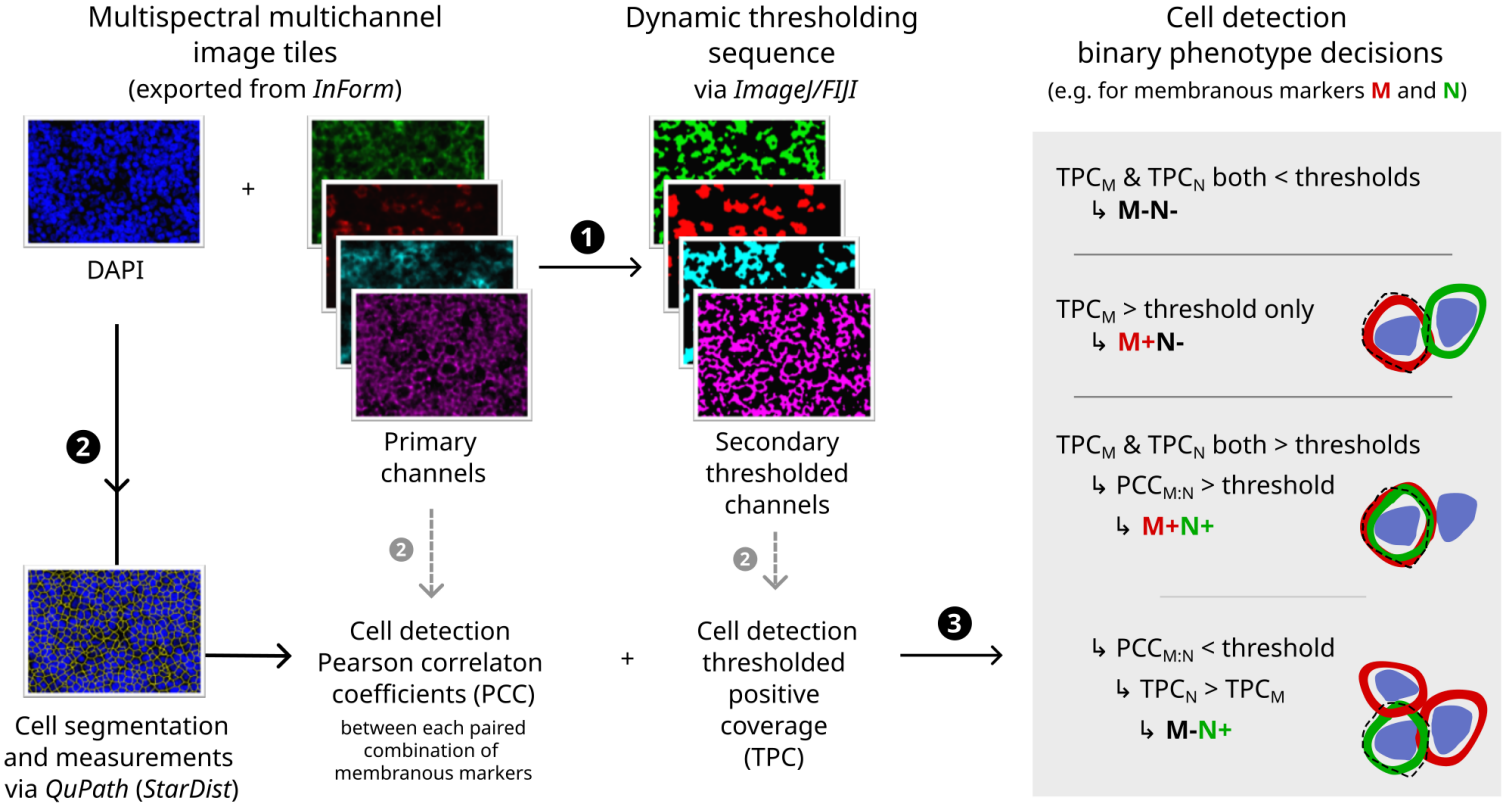
Digital image analysis and cell phenotyping workflow. Flowchart indicating major steps (1–3) with illustrative primary immunofluorescence image channels (left), secondary thresholded channels (middle) and diagrammatic rationale for phenotyping decisions (right; dashed line = outline of cell image segment derived computationally by expansion of nuclear detection segmentation). For further details and additional example fields, see **Supplementary Methods** and **Supplementary Figures S1**, **S2**.

The subsequent image analysis workflow employed non-hierarchical binary phenotyping performed in open-source software, outlined in **Figure 2** and further detailed in **Supplementary Methods**. Of note, phenotyping was augmented by two innovative image processing techniques: (i) dynamic image thresholding, which minimised sensitivity to technical variations in signal/background characteristics (**Supplementary Figure S1**); and (ii) colocalisation-based resolution of cells with provisional positivity for multiple membranous markers, which minimised spurious phenotypes arising due to contaminating signals between immune cell membranes in the crowded TME of NLPHL/THRLBCL (**Supplementary Figure S2**).

Fractions of immune cell phenotypes in image tiles were compared between case types using Mann-Whitney U tests due to non-normally distributed data. In lymphocyte panel images, nearest-neighbour distances between LP cells and non-neoplastic lymphocyte phenotypes were also compared. P-values were expressed with Bonferroni adjustment and *p*<0.001 regarded as significant.

## Results

### Workflow validation and interpretation of differential phenotype frequency

The phenotyping workflow provided accuracy of 0.95-0.99 in determining appropriate provisional binary positive/negative status for each individual marker. Colocalisation-based resolutions of cells provisionally positive for two membranous markers provided accuracy of 0.71-0.87 in distinguishing convincing calls from likely spurious calls, which was superior to accepting all provisionally double-positive cells as genuine. Precision across different multiplexing permutations was demonstrated by a strong correlation (*R*=0.98) between CD8-positive cell fractions in matched tissues stained with the lymphocyte versus cytotoxic/NK panels. The workflow validation is further detailed in **Supplementary Results** and exemplified by **Supplementary Figures S3**, **S4**.

**Figure 3:**
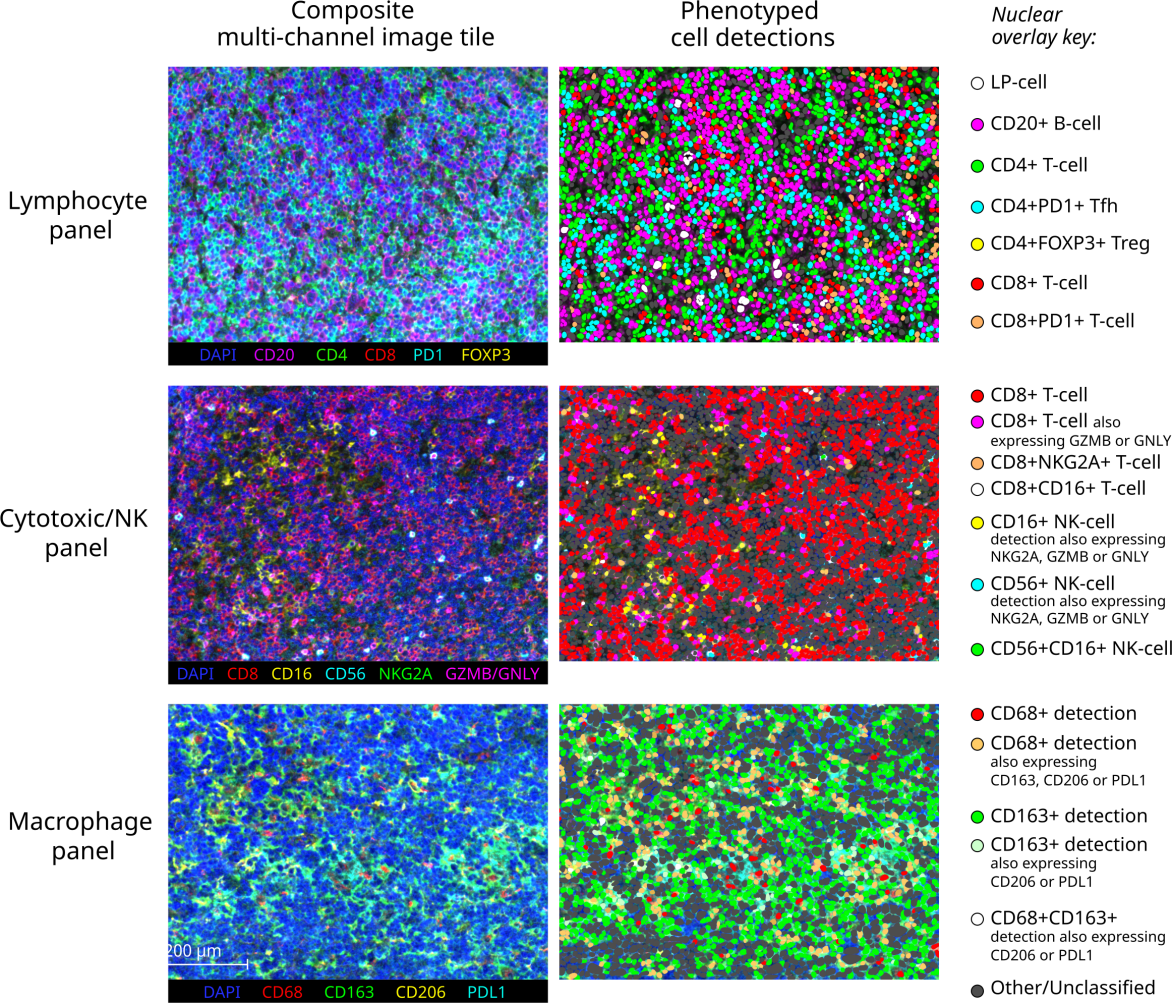
Exemplary multichannel image tiles and major immune cell phenotypes. Representative 670x501 micron multispectral immunofluorescence image tiles from a pattern E NLPHL case (left) stained with each of the three panels (each row), with corresponding cell detections illustrated as nuclear overlays colour-coded by derived phenotype group (right).

Major resulting derived phenotypes from each panel are mapped to representative multi-channel image tiles from an exemplary NLPHL-pE case in **Figure 3**. To account for the expected differences in overall B-cell to non-B-cell ratio which typifies NLPHL histological patterns and THRLBCL, quantities of differentially increased/decreased phenotypes were compared as fractions relative to a parent population (summarised in **Table 2** and further presented in following sub-sections).

**Table 2:** Differentially expanded or depleted populations between case types.

	Case type					
	Tonsil	NLPHL-pA	NLPHL-pC	NLPHL-pE	THRLBCL	LRcHL
Lymphocyte panel T-cell phenotypes: median percentage of total CD20-negative cells						
**CD4+FOXP3+**	3.8%	0.8% ↓^T/L^	0.6% ↓ ^T/L^	0.4% ↓ ^T/L^	N/A †	6.0%
**CD4+PD1+**	3.4%	11%	12%	6.0% ↓^A,C^	13%	7.4%
**CD8+PD1+**	0.4%	2.6%	2.0%	7.5% ↑^A,C^	12% ↑↑^A,C,E^	2.5%
**CD4+CD8+PD1+**	0.00%	0.02%	0.01%	0.05% ↑^A^	0.40% ↑↑^E^	0.07%
Cytotoxic panel NK-cell phenotypes: median percentage of total NK-cell population*						
**CD56+NKG2A+**	49%	46%	39%	9.3% ↓^A,C^	0.8% ↓↓^A,C,E^	0.0%
**CD16+GZMB+**	8.6%	16%	36% ↑^A^	64% ↑↑^A,C^	61% ↑↑^A,C^	11%
Cytotoxic panel CD8+ T-cell phenotypes: median percentage relative of total CD8+ cells						
**CD8+NKG2A+**	0.6%	1.2%	1.4%	1.0% ↓^C^	0.2% ↓^A,C,E^	0.1%
**CD8+CD16+**	0.0%	0.0%	0.3% ↑^A^	1.1% ↑↑^C^	4.0% ↑↑^A,C,E^	0.0%
**CD8+GZMB+**	1.1%	1.6%	2.4%	9.0% ↑^C^	11% ↑^C^	2.5%
Macrophages panel phenotypes: median percentage of total CD68+ and/or CD163+ cells						
**CD163+**	49%	42%	62%	73% ↑^A^	82% ↑^A^	26%
**CD163+PDL1+**	0.3%	1%	6% ↑^A^	11% ↑^A^	24% ↑↑^A,C,E^	1%
**CD68+**	63%	68%	49%	40% ↓^A^	42% ↓^A^	84%

NLPHL, nodular lymphocyte predominant Hodgkin lymphoma; -pA, pure pattern A; -pC: pattern C dominant; -pE, pattern E dominant; THRLBCL, T-cell/histiocyte-rich large B-cell lymphoma; LRcHL, lymphocyte-rich classical Hodgkin lymphoma.

↑/↓: significant increase/decrease (Mann Whitney U test p<0.001) between case types indicated by superscript as follows. T/L: compared to tonsil and LRcHL; A: compared to NLPHL-pA; C: compared to NLPHL-pC; E: compared to NLPHL-pE.

**Figure 4:**
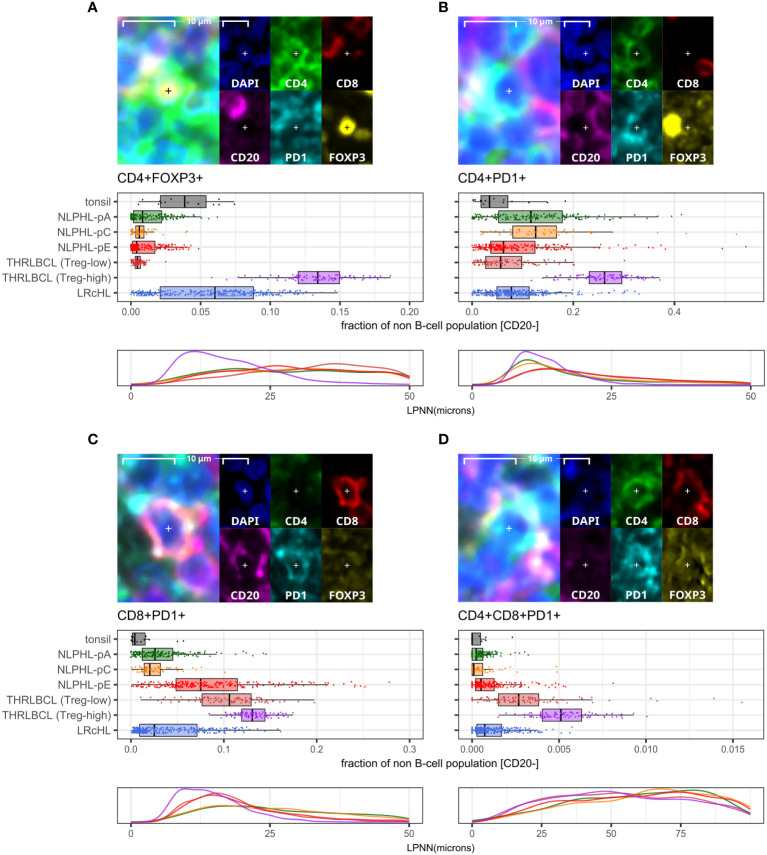
Differential T-cell phenotypes derived via the lymphocyte panel. For each phenotype shown **(A–D)**: representative detection (upper; multispectral fluorescence composite image [left] and component channels [right]); phenotype fractions in image tiles by case type, quantified relative to the parent non-B cell population (middle); and LP-to-phenotype nearest neighbour (LPNN) distribution by case type (lower; hd, histogram density).

† Median not reported due to bimodal heterogeneity between cases (see results text and **Figure 4A**).

* NK-cell defining criteria: CD8- AND [CD16+ AND/OR CD56+] AND [NKG2A+ AND/OR GZMB+ AND/OR GNLY+].

Of note, we report a marker (M) status binarily as positive (+) or negative (-). Our “M+” phenotypes highly express M and likely best correspond to M^high^/M^bright^ as reported by other methods continuously quantifying signal intensity, such as flow cytometry. Meanwhile, our “M-” phenotype may correspond to either M- or M^low^/M^dim^ as reported by such methods. We were cognisant of this caveat when considering the presence of established biological phenotypes characterised by bright/dim marker expression.

### CD4+FOXP3+ T-regulatory cells are uniformly depleted in NLPHL patterns A, C and E

CD4+FOXP3+ cells were uniformly reduced in all NLPHL cases (median 0.3-0.7% of non-B cells) in comparison to LRcHL cases and tonsil controls (4-6%; **Figure 4A**). These CD4+FOXP3+ cells are likely to represent T-regulatory cells (Tregs), which have also been found to be depleted in corroborative flow cytometry-based studies (21, 22). In contrast, THRLBCL cases showed heterogeneity in CD4+FOXP3+ cell fractions. Three ‘Treg-low’ TRHRLBCL cases had levels comparable to NLPHL and one ‘Treg-high’ THRLBCL case had levels greater than LRcHL cases and tonsil controls.

We also observed that, in comparison to the ‘Treg-low’ THRLBCL and NLPHL cases, the single Treg-high THRLBCL case showed increased CD4+PD1+ Tfh cells, CD4+FOXP3+PD1+ cells, and B-cells (**Figure 4B**; **Supplementary Figure S5**).

### CD8+ T-cells in NLPHL pattern E and THRLBCL are depleted of an NKG2A+ population and show expansion of CD16+, GZMB+, and PD1+ populations

**Figure 5:**
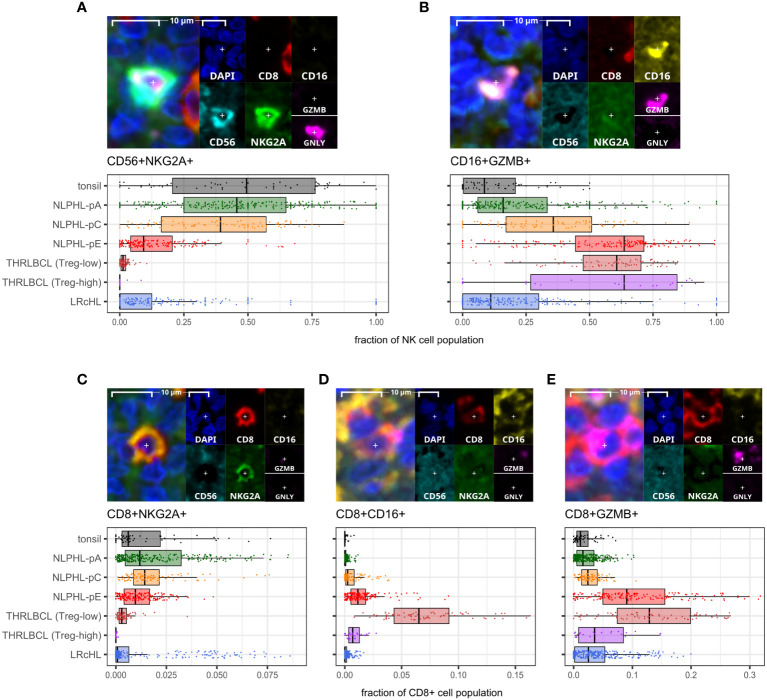
Differential NK and CD8+T cell phenotypes derived via the cytotoxic/NK panel. For each phenotype shown **(A–E)**: representative detection (upper; multispectral fluorescence composite image [left] and component channels [right]); and phenotype fraction in image tiles by case type, quantified relative to the parent total NK-cell or total CD8+ T-cell populations, as indicated (lower). NK-cell defining criteria were: CD8- AND [CD16+ AND/OR CD56+] AND [NKG2A+ AND/OR GZMB+ AND/OR GNLY+].

A minor population of CD8+NKG2A+ cells showed a peak in NLPHL-pC cases (median 1.4% of CD8+ cells) followed by significant depletion in NLPHL-pE cases (1%) which progressed further in THRLBCL cases (0.2%; **Figure 5C**).

In contrast, compared to NLPHL-pA/pC cases, NLPHL-pE and THRLBCL cases showed increased CD8+GZMB+ cells (median 9% and 11% of CD8+ cells, respectively; **Figure 5E**); CD8+CD16+ cells (1% and 4%; **Figure 5D**); and CD8+PD1+ cells (8% and 12% of non-B-cells; **Figure 4C**). The CD8+GZMB+ cells likely represent activated cytotoxic T-cells, whilst the CD8+CD16+ cells may represent a small population of activated innate cytotoxic T-cells with NK-like function (23). The CD8+PD1+ cells may either represent recently activated cytotoxic T-cells with transiently increased PD1 or functionally exhausted CD8+ T-cells (24, 25). The combined findings are overall suggestive of ongoing or antecedent adaptive T-cell tumour-reactive immune responses in NLPHL-pE and THRLBCL cases.

CD8+PD1+ T-cells were also spatially closer to tumour cells in NLPHL-pE and THRLBCL cases, compared to NLPHL-pA/C cases (**Figure 4C**; nearest neighbour distance plots). In these cases, we visually confirmed that CD8+/CD8+PD1+ T-cells directly contacted tumour cells but did not form phenotypically homogenous circumferential rosettes. Circumferential CD4+PD1+ Tfh-cell rosettes were only present in NLPHL-pA cases and in the nodular B-cell rich component of NLPHL-pC/E cases. In contrast, when T-cells surrounded tumour cells in T-cell rich areas of NLPHL-pC/E and THRLBCL cases, they were occasionally all PD1 positive (and may have been labelled as “PD1-positive rosettes” by singleplex methods) but they were of heterogenous CD4+ or CD8+ phenotypes reflective of the wider microenvironment rather than a localised *bona fide* rosette.

CD4+CD8+PD1+ cells represented a very minor sub-population (0-0.5% in NLPHL cases) with similar differential representation between case types as its parent CD8+PD1+ population (**Figure 4D**).

### NK phenotypes progressively shift from CD56+NKG2A+ to CD16+GZMB+ as histology progresses from NLPHL pattern A to C to E and to THRLBLCL

CD16+GZMB+ cells were progressively expanded between NLPHL cases: proceeding from a low in NLPHL-pA cases (median 16% of all NK-cells), rising in NLPHL-pC cases (36%) and reaching a plateau between NLPHL-pE and THRLBCL cases (61% and 64%). In parallel, CD56+NKG2A+ cells (including a minor GNLY+ subset) were depleted in NLPHL-pE cases (9.3%) and THRLBCL (0.8%), compared to NLPHL-pA/C cases (46%/39%; **Figures 5A, B**).

With reference to established NK-cell immunobiology, this phenotypic switch appears consistent with expansion of tumour-reactive activated effector CD16^bright^CD56^dim^ NK-cells by maturation from the pool of nodal CD56^bright^CD16^dim^ NK-cells undergoing education and licensing of “self”-receptors such as NKG2A (26, 27).

### Predominant macrophage populations shift from CD68+ to CD163+ with an increasing PDL1+ sub-population as histology progresses from NLPHL pattern A to C to E

**Figure 6:**
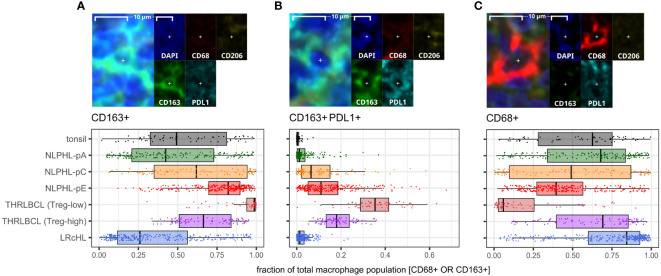
Differential macrophage detection phenotypes derived via the macrophage panel. For each phenotype shown **(A–C)**: representative detection (upper; multispectral fluorescence composite image [left] and component channels [right]); and phenotype fractions in image tiles by case type, quantified relative to the parent total macrophage population (lower).

CD163+ macrophage detections, including an increasingly prominent PDL1+ subpopulation, were progressively expanded from a low in NLPHL-pA cases (median 42% of total CD68 and/or CD163 positive detections) to a high in NLPHL-pE cases (73%); whilst CD68+ detections apparently became comparatively depleted (**Figure 6**). CD206+ macrophage detections were much rarer (<1%) and not meaningfully differentially represented between case types. Only a minority of macrophage detections appeared CD68+CD163+ double-positive, some of which were also PDL1+ (**Supplementary Figure S6**).

The apparently limited co-expression of CD68 and CD163 was not expected. In some morphological macrophage detections, CD68 signal appeared genuinely low/near-negative, possibly reflecting macrophages with diminished phagocytic function. However, CD163+ macrophages were often noted to have moderate/abundant cytoplasm with dendritic-like processes, leading to CD163’s membranous signal being distant from DAPI’s nuclear signal and CD68’s perinuclear cytoplasmic signal. The limited co-expression may therefore be partly methodological due to the nuances of digitally segmenting such cells. Regardless, we regard CD163+ detection proportions as a meaningful surrogate for macrophage phenotypes in our case repertoire, given the significant differential findings and frequent colocalisation with PDL1.

## Discussion

### Role of Tfh cells and exclusion of Tregs in NLPHL lymphomagenesis

The depletion of CD4+FOXP3+ cells across all NLPHL cases (including Fan histological patterns A, C and E in this study) points to a TME signature necessary for lymphomagenesis. NLPHL development has been shown to be related to chronic stimulation by driver antigens in a Tfh-dependent manner (28). This may include exogenous microbial antigens as exemplified by Thurner et al. (29).

We suggest that exclusion of CD4+FOXP3+ cells is a tumour-permissive requirement mandatory for the maintenance of tumour-driving chronic antigenic stimulation and LP : Tfh cell interactions. In keeping with this concept, the depleted CD4+FOXP3+ population has been shown by flow cytometry to include both Tregs and T-follicular regulatory (Tfr) cells (22). Both are known to regulate and suppress B:Tfh interactions in states of infection-related antigenic stimulation (30).

Whilst the initial restriction of LP cells in typical indolent cases of NLPHL to nodular microenvironments rich in B-cells and Tfh could be in part explained by a direct reliance of LP cells on Tfh interactions, we suggest that the nodular microenvironment may also provide a relative immune-privileged “shielding” effect.

### Evidence of progressive anti-tumour immune responses followed by a tolerogenic microenvironment across the histological spectrum of NLPHL and THRLBCL

The concept of an immune-privilege effect mediated by B-cell/Tfh-rich nodular environments is supported by our finding of an innate effector NK-cell response coinciding with LP cells beginning to reach beyond nodules (Fan histological pattern C). The importance of functional NK cells in NLPHL surveillance is corroborated by the observation of NLPHL development in siblings with genetic defects in NK cell development (31). Additionally, an adaptive CD8+ T-cell response became apparent when LP cells were found in more diffusely extra-nodular environments (Fan histological patterns E and THRLBCL), and this was associated with dismantling of circumferential Tfh-cell rosettes.

We found that these NK/T-cell immune responses coincided with relative depletion of NKG2A-positive NK-cells and CD8+ T-cells, suggesting an early role of NKG2A loss/downregulation in permitting anti-tumour responses. The role of NKG2A:HLA-E axis signalling is well established in NK-cell responses and more recently established in a subset CD8+ T-cells mediating non-classical HLA-E restricted responses (32).

The expansions of activated NK-cell and CD8+ T-cell populations in THLRBCL and pattern E dominant NLPHL cases coincided with progressive expansions of CD163+ and CD163+PDL1+ macrophage and likely exhausted CD8+PD1+ T-cell populations. These populations may reflect the compensatory mechanisms that maintain an overall net tumour-permissive TME.

The macrophage detections in pattern E dominant NLPHL cases were increasingly CD163-positive and often CD68-low or apparently negative. Our experience with macrophage markers in NLPHL and THRLBCL cases in this study supports superiority of CD163 over CD68 as a macrophage marker of relevance and increased expression in NLPHL with variant patterns, and is corroborated in a larger cohort by conventional immunohistochemistry (6). CD163+PDL1+ macrophages were markedly increased in THRLBCL cases, which may represent alternative activation of macrophages with tumour-promoting immuno-suppressive function via the PD1:PDL1 immune checkpoint axis. This is corroborated by other published studies including transcriptomic evidence of a more tolerogenic TME in THRLBCL compared to NLPHL and immunophenotypic evidence of a TME rich in PD1:PDL1 interations in THRLBCL (33–35).

### THRLBCL cases may develop heterogenous or divergent tolerogenic mechanisms

THRLBCL cases showed dichotomous heterogeneity in fractions of some TME immune cell phenotypes, particular CD4+FOXP3+ Tregs and CD4+FOXP3+PD1+ Tfr-like cells. The Treg-low THRLBCL cases show further expansion of the same immune cells types as seen in pattern E dominant NLPHL. This supports the concept that these cases reflect progression from NLPHL.

In contrast, the single Treg-high THRLBCL case showed a divergent TME with increased Tregs, Tfr-like cells, and Tfh; accompanied by a marked reduction in activated CD8+GZMB+ T-cells. We suggest this indicates that the aforementioned requirement of Treg exclusion may not be powerful sculptor of the TME in this particular THRLBCL case, and that the acquisition of Tregs/Tfr-like cells may confer more potent immune tolerance.

Whilst the heterogeneity of the TME in THRLBCL may correlate to different states of lymphoma progression, another possibility is that a divergent Treg-high TME in THRLBCL cases may represent *de novo* cases not progressed from NLPHL. In any case, we suggest a larger clinically-correlated cohort study is warranted to explore the utility of Treg/Tfr-like cell quantification as a clinically relevant marker in THRLBCL.

### Speculations on the nature of CD4+CD8+PD1+ double-positive T-cells in NLPHL

Multiple flow cytometry studies report CD4+CD8^dim^PD1^bright^ double-positive T-cell (DPT) populations as a feature with relative specificity for the NLPHL TME (22, 36–38). However, we note that our CD4+CD8+PD1+ population was present in NLPHL cases at fractions similar to or lower than those of LRcHL (i.e., lacking specificity for NLPHL) and orders of magnitude lower than the flow-defined CD8^dim^ DPT cells. The trace CD4+CD8+PD1+ population we detected is therefore likely to, at most, only partially overlap with flow-defined DPT populations.

We note that a detailed characterisation of DPTs in NLPHL reveals a phenotype similar to Tfh cells (CD4+, CCR7-, PD1^bright^, CXCR5+, TIGIT+, CTLA4+) with the addition of dim CD8 (22). We speculate that the DPTs in NLPHL could represent Tfh cells which have non-transcriptionally acquired trace CD8 molecules through trogocytosis. If so, the DPT population can be expected to be more readily detectable by flow cytometry than by cross-sectional histology relying on uniform membranous expression. We can further speculate that LP cells forming immunological synapses with both Tfh cells and functionally inhibited tumour-reactive CD8+ T-cells sets the stage for the prolonged contact between these three cells, and that this may facilitate such a transfer of CD8 molecules to Tfh/Tfh-like cells.

### Concluding remarks

In this study, we developed an innovative multiplexed immunofluorescence and digital analysis workflow to generate millions of phenotyped cell detections from hundreds of histological images derived from routine biopsy material. Our results corroborate and further characterise key alterations in the TME of NLPHL and THRLBCL.

**Figure 7:**
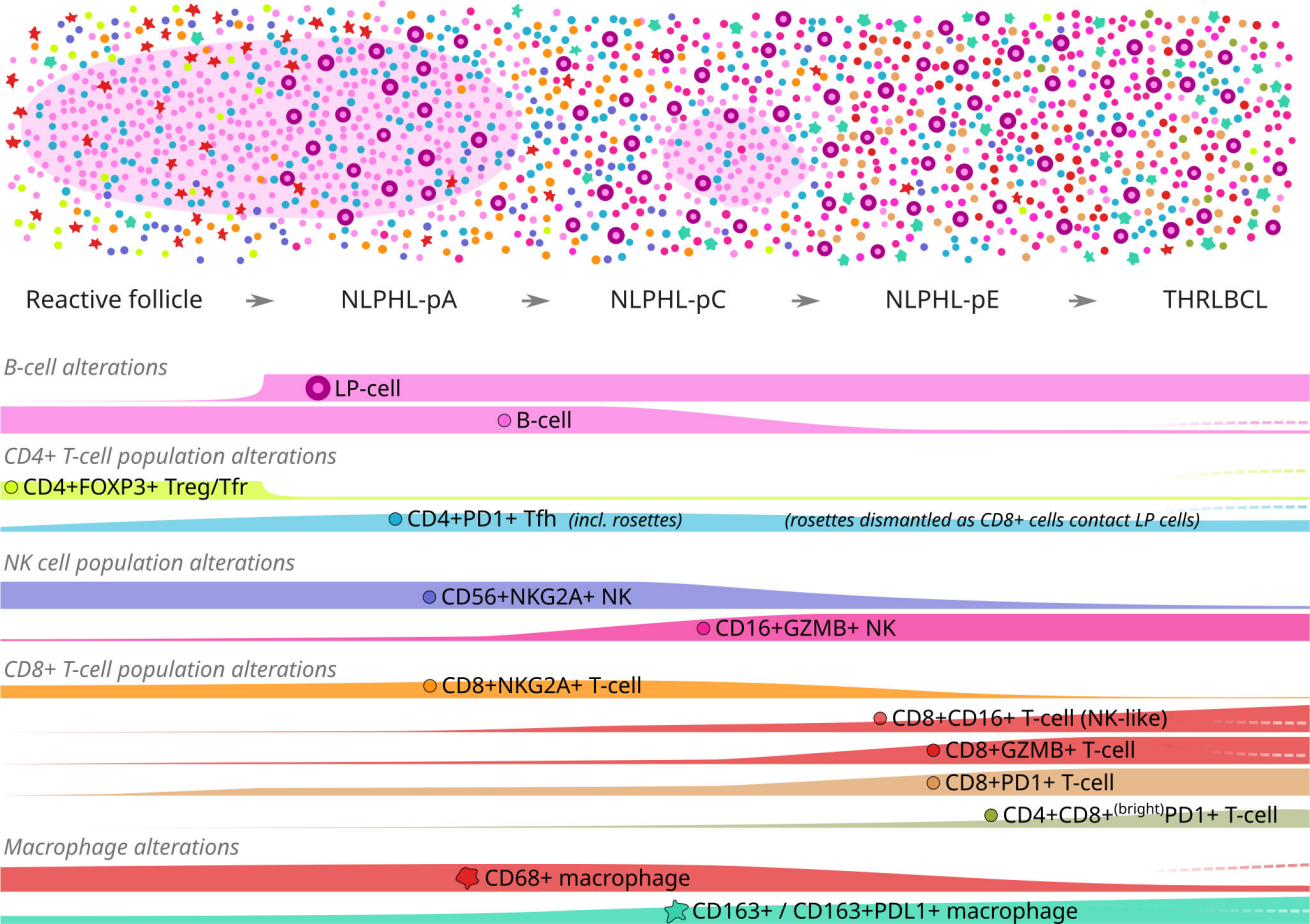
Graphical summary of differential microenvironmental phenotypic populations mapped to an idealised NLPHL/THRLBCL continuum. Illustration of cellular tumour composition (above) and phenotype mountain plots (below) for each phenotype of interest. Illustration key: light pink ovoids = B-cell rich nodules; cell phenotypes otherwise coloured/shaped as per icons in mountain plot labels. Mountain plots are illustrative only, based on relative findings between case types, and are not to scale; dashed lines (right) indicate the alternative alterations seen in the single “Treg-high” THRLBCL case.

We demonstrate progressive shifts in TME immune cell phenotypes between histological patterns of NLPHL and THRLBCL (illustrated in **Figure 7**), which provide further support for a *bona fide* biological continuum between these lymphomas. As we have reported these phenotypes in simple binary terms, their detection may be amenable to dual-chromogenic staining protocols and simplified analysis, facilitating more translatable quantification in larger clinically correlated cohorts as potential biomarkers of high-risk disease.

Further biological studies assessing any molecular features of the neoplastic LP cells which accompany the changes in NLPHL patterns and microenvironmental populations may provide further support of a continuum between NLPHL and THRLBCL, as well as possible mechanistic explanations for progression along the continuum. Such studies might include assessment of PDL1/PDL2 gene gains and protein expression in tumour cells, which are noted to be absent in LP cells of typical NLPHL cases but present in THRLBCL tumour cells (35, 39, 40).

Meanwhile, our findings permit speculation that the immuno-biology of the NLPHL-THRLBCL continuum involves three major immunoregulatory mechanisms: (i) exclusion of Treg/Tfr-mediated regulation of Tfh function in NLPHL; (ii) possible early NKG2A:HLA-E axis mediated regulation of cytotoxic NK and CD8+T cell responses in pattern A and C NLPHL; and (iii) a PD1:PDL1 axis mediated immune checkpoint between exhausted CD8+PD1+ T-cells and PDL1+ macrophages accompanied by dismantling of homogenous Tfh-cell rosettes, which increasingly defines the TME with progression to NLPHL pattern E and to THRLBCL.

The relevance of these immune checkpoint axes builds on the rationale for their therapeutic interruption in NLPHL and THRLBCL. Lessons from anti-PD1 therapy in classical Hodgkin lymphoma show that high expression of the checkpoint co-inhibitory receptor (i.e., PD1) on T-cells does not predict treatment response, and that responses are mediated by permitting recruitment of new tumour-reactive T-cells to a TME enriched in the co-inhibitory ligand (41). By a similar logic, our finding of depleted NKG2A-expressing NK and T-cells in NLPHL pattern E and THRLBCL need not discourage the relevance of the NKG2A:HLA-E axis. Anti-NKG2A therapy shows promise in solid tumours as monotherapy or in combination with anti-PD1 therapy (42). We suggest that, if anti-NKG2A therapy continues to become established, quantification of co-inhibitory ligand expression for both axes (HLA-E and PDL1) may become useful predictive biomarkers when considering single or combined immunotherapy in high-risk NLPHL and THRLBCL.

## Data availability statement

The raw data supporting the conclusions of this article will be made available by the authors, without undue reservation.

## Ethics statement

The studies involving humans were approved by University College London Research Ethic Committee (Approval title: Immunohistochemical and molecular studies of human lymphoma – an alternative approach for the identification of new biomarkers; 09/H0715/64). The studies were conducted in accordance with the local legislation and institutional requirements. The human samples used in this study were acquired from a by- product of routine care or industry. Written informed consent for participation was not required from the participants or the participants’ legal guardians/next of kin in accordance with the national legislation and institutional requirements.

## Author contributions

CP: Data curation, Funding acquisition, Investigation, Methodology, Software, Validation, Visualization, Writing – original draft, Writing – review & editing. AA: Investigation, Methodology, Project administration, Software, Validation, Writing – review & editing. AR: Writing – review & editing. AS: Writing – review & editing. BF: Writing – review & editing. MP: Writing – review & editing. DL: Writing – review & editing. TM: Conceptualization, Funding acquisition, Project administration, Resources, Supervision, Writing – review & editing.
