# Supplementary material for: Microenvironmental immune cell alterations across the spectrum of nodular lymphocyte predominant Hodgkin lymphoma and T-cell/histiocyte-rich large B-cell lymphoma
Source: Front Oncol. 2023 Oct 3;13:1267604. doi: 10.3389/fonc.2023.1267604 (PMC10579566; doi:10.3389/fonc.2023.1267604)
Supplement: Supplementary file 1 [file DataSheet_1.pdf]

Supplementary Methods

Antibodies and fluorophores for multiplexed immunofluorescence

TARGET	CLONE	VENDOR	DILUTION	PAIRED FLUOROPHORE*
Lymphocyte panel				
**CD4**	4B12 (M)	Leica	1:50	Opal 520
**CD8**	4B11 (M)	Leica	1:200	Opal 620
**CD20**	L26 (M)	Agilent	1:200	Opal 690
**PD1**	NAT 105/E3 (M)	Abcam	1:350	Opal 650
**FOXP3**	236A/E3 (M)	Abcam	1:200	Opal 540 or Opal 570
Cytotoxic/NK panel				
**CD8**	4B11 (M)	Leica	1:200	Opal 650
**CD16**	2H7 (M)	Leica	1:40	Opal 570
**CD56**	CD564 (M)	Leica	neat	Opal 520
**NKG2A**	EPR23737-127 (R)	Abcam	1:1000	Opal 690
**Granzyme-B**	11F1 (M)	Leica	1:40	Opal 540
**Granulysin**	F-9 (M)	Santa Cruz	1:300	Opal 620
Macrophage panel				
**CD68**	PGM1 (M)	Agilent	1:100	Opal 520
**CD163**	10D6 (M)	Leica	1:200	Opal 690
**CD206**	Rabbit polyclonal	Abcam	1:250	Opal 620
**PDL1**	22C3 (R)	Agilent	neat	Opal 570

(M)= Mouse monoclonal; (R)= Rabbit monoclonal; Leica= Leica Microsystems Ltd., Newcastle-upon-Tyne, UK; Agilent= Agilent Technologies LDA UK Ltd., Cheshire, UK; Abcam= Abcam Plc., Cambridge, UK; Santa Cruz= Santa Cruz Biotechnology Inc., Texas, USA;
* Fluorophores from the 'Opal 7-Color' kit (Akoya Biosciences, Marlborough, MA, USA)

Digital image analysis workflow (1): Dynamic image thresholding

Dynamic pixel thresholding was executed in 'ImageJ' (FIJI distribution; version 1.53q) to generate secondary thresholded channels with relative insensitivity to wider technical variations in signal/background characteristics.

Functions were executed in the following sequence (with further rationales for their inclusion provided):

1. "*Despeckle*" on *primary channels* for all markers (not including DAPI).
   - Rationale: this de-noising function helped to make generate more informative Pearson correlation coefficient measurements between channel pairs (see following sections).

2. "*Duplicate"* on *primary channels* to generate *secondary channels.*
3. "*Enhance Local Contrast (CLAHE)*" on *secondary channels* with settings: blocksize=25; histogram=100; maximum=4.5; fast mode.
4. "*8-bit*" conversion on *secondary channels.*
   - Rationale: required to ensure consistent Auto Threshold behaviour.
5. "*Auto Threshold*" on *secondary channels* with settings: method=RenyiEntropy; ignore white; white objects on black background.
   - Rationale: to generate *secondary thresholded channels* with binary pixel values of either zero (in negative background) or 255 (in positive signal).
   - Note: this is the 'plugin' and not the 'applet' implementation of FIJI's thresholding functions.
6. "*Close*" binary function on *secondary thresholded channels* with settings: iteration=1; count=2.
   - Rationale: this partially fills concave or ring-like thresholded objects, thickening the inner aspect of membranous signals and thus augmenting the subsequent detection of truly positive cells.
7. "*Remove Outliers*" binary function on *secondary thresholded channels* with settings: radius=2; threshold=1.
   - Rationale: to remove speck-like pixel outliers.
8. "*Divide"* math function on *secondary thresholded channels* with value = 255.
   - Rationale: this changes to binary pixel values to either zero or 1, meaning than mean pixel values in subsequent cell detections become equivalent to coverage by thresholded positive signal.

   NB:: "*resetMinAndMax()*" was also executed between each step to ensure consistent behaviour of functions, because some can be affected by alterations to the Look Up Table minima and maxima.

Two human investigators (CP/AA) independently quality-checked all outputs from this sequence. Tiles generating a non-satisfactory output secondary thresholded channel (i.e., excessively under or over-thresholded) for any marker were excluded (example satisfactory and non-satisfactory outputs shown in Figure S1).

Digital image analysis workflow (2): Cell segmentation and measurements

Processed images with both including both primary channels and secondary threshold channels were then segmented using 'StarDist' (version 0.3.0), a deep-learning model

implemented in ‘QuPath’ (version 0.3.0), to generate single-cell detections. The script parameters were as follows:

- StarDist settings:
  - Model name = dsb2018_heavy_augment.pb*
  - Probability (detection) threshold = 0.5
  - Pixel size = 0.3
  - Cell expansion = 3.0
  - Cell constrain scale = 2.0
  - Ignore cell overlaps = FALSE
  - Simplify = 1
  - Measure shape
  - Measure intensity
- Additional appended sub-scripts
  - ID-tagging script (to enable later cross-referencing and visual correlation with phenotypes derived in other software)
  - Pearson correlation coefficient (PCC) measuring scripts** for each paired combination of membranous markers in the panel (to measure colocalisation at the individual cell detection level)

* Pre-trained model available at https://github.com/qupath/models/tree/main/stardist [accessed March 2023]

** Script adapted from a thread posted publicly on ‘image.sc’ https://forum.image.sc/t/if-colocalization-calculations-for-qupath-updated-to-0-3-2/25165 [accessed March 2023]; PCC was measured in whole-cell compartments between paired primary channel pixel values.

Two human investigators (CP/AA) independently manually quality-checked all outputs after segmentation and regions with gross non-cellular under/over-staining artefacts were excluded. Additionally, detections with extremes of nuclear diameter (<4 microns or >11 microns, representing over/under-segmented nuclear detections respectively) were excluded from subsequent phenotyping. In image tiles stained with the lymphocyte panel, CD20-positive LP cells were manually annotated to distinguish them from non-neoplastic B-cells in the subsequent analyses.

Digital image analysis workflow (3): Algorithmic phenotyping

RStudio (version 1.4.1717, running R 4.11) was used to run algorithmic phenotyping and further analysis. Phenotypes of cell detections were primarily determined via thresholded positive coverage (TPC; measured in the whole-cell compartment via secondary thresholded channels). TPC determined the final status for nuclear (FOXP3) and cytoplasmic-predominant

(GZMB/GNLY/CD68) markers. However, if detections were provisionally positive for two membranous markers based on TPCs, Pearson correlation coefficients (PCC) between primary channel intensities were implemented as an additional accuracy-improving step in the context of a crowded TME. The rationale being that high PCC can differentiate double-positive phenotypes with colocalising signals from those with low PCC which are likely spurious phenotypes (due to contamination of cell detections by neighbouring cell membranes), and that the difference between TPCs can determine which is the likely contaminant (illustrated in Figure S2). The optimised TPC and PCC base thresholds applied are provided below.

Detections appearing positive for three or more membranous markers were similarly resolved by consensus agreement of pair-wise sub-resolutions (i.e., if a marker is resolved as genuinely positive in all the required pair-wise resolutions it features in, it is considered positive by consensus).

Fixed thresholds applied in algorithmic phenotyping

	Lymphocyte panel		*Cytotoxic/NK panel*		*Macrophage panel*	
TPC thresholds	CD4	0.18	CD8	0.25	CD68	0.15
	CD8	0.25	CD16	0.25	CD163	0.35
	CD20	0.32	CD56	0.35	CD206	0.2
	PD1	0.25	NKG2A	0.25	PDL1	0.2
	FOXP3	0.2	Granzyme-B	0.15	-	-
	-	-	Granulysin	0.18	-	-
PCC thresholds	CD4:CD8	0.4	CD8:CD16	0.3	CD163:CD206	0.2
	CD4:CD20	0.8	CD8:CD56	1*	CD163:PDL1	0.2
	CD4:PD1	0.1	CD8:NKG2A	0.25	CD206:PDL1	0.2
	CD8:CD20	0.7	CD16:CD56	0.15	-	-
	CD8:PD1	0.3	CD16:NKG2A	0.15	-	-
	CD20:PD1	1*	CD56:NKG2A	0.15	-	-
'Difference in TPC' thresholds	CD4–CD8	0	CD8–CD16	0.05	CD163–CD206	0
	CD4–CD20	-0.1	CD8–CD56	-0.15	CD163–PDL1	0.15
	CD4–PD1	-0.1	CD8–NKG2A	0.1	CD206–PDL1	0
	CD8–CD20	0	CD16–CD56	-0.1	-	-
	CD8–PD1	0	CD16–NKG2A	0	-	-
	CD20–PD1	0	CD56–NKG2A	0.3	-	-

TPC = thresholded positive coverage; PCC = Pearson correlation coefficient.

* PCC thresholds of 1 indicate that no visually convincing double-positive cells of that kind were identified in representative evaluation sets.

Supplementary Results

Phenotyping workflow evaluation

To evaluate the accuracy of the phenotyping workflow, an evaluation subset of cell-detections stratified to cover a full range of TPC and PCC values and cases was randomly generated, in which groundtruth was then established by visual manual classification. TPC thresholds imparted accuracy of 0.95-0.99 in determining appropriate positive/negative status for all markers (exemplified in Figure S3A-B). PCC-based resolutions provided accuracy of 0.71-0.87 in distinguishing convincing from likely spurious double-positivity (exemplified in Figure S4).

To validate the precision of the workflow, cropped image areas from matching tissue regions stained with the different lymphocyte and cytotoxic/NK panels were obtained (based on available stromal/edge landmark profiles). The correlation of CD8+ detections derived from each panel (R=0.98) is shown in Figure S3C.

Supplementary Figures

Figure S1: Example outputs from dynamic image thresholding sequence

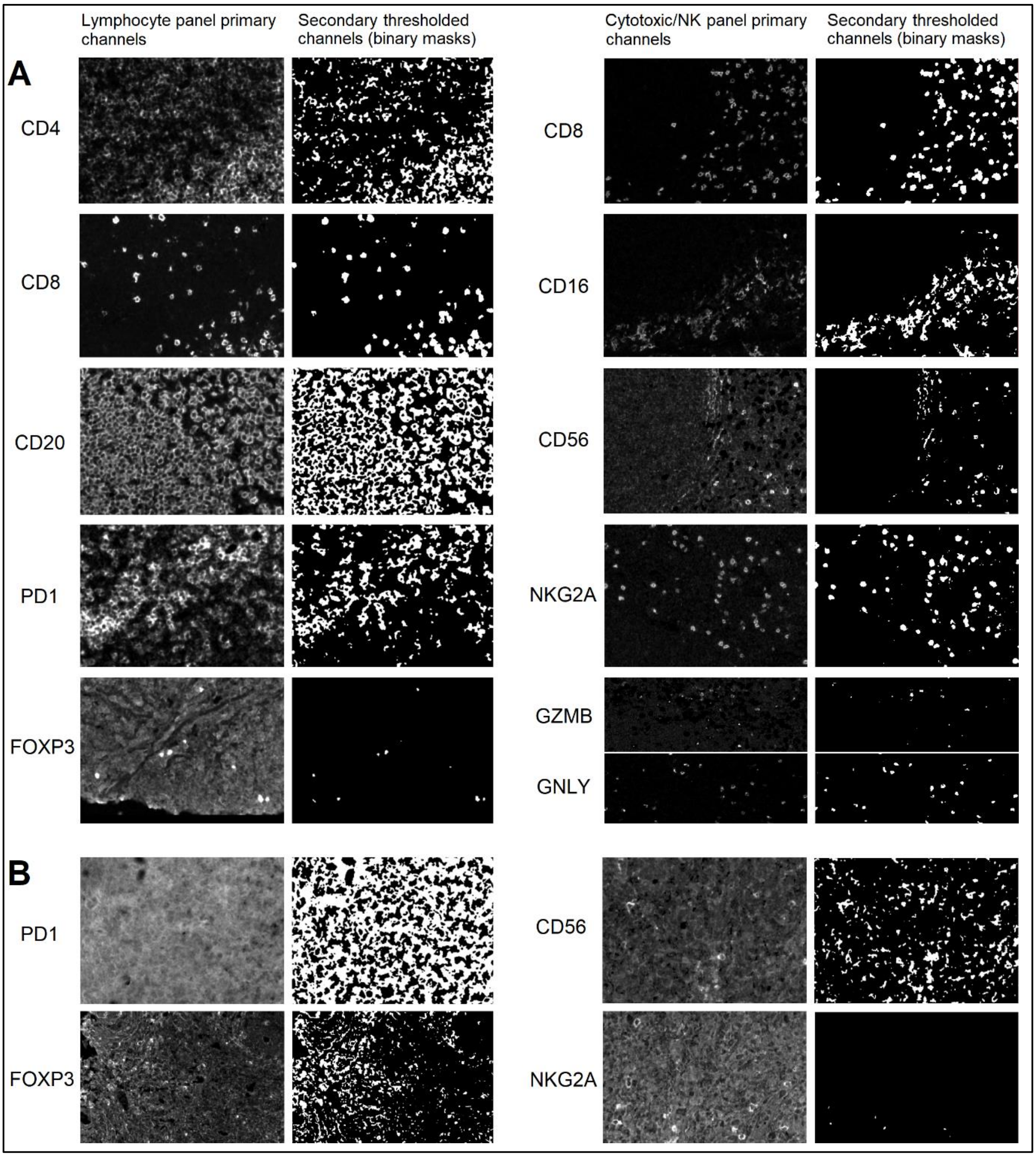

Legend: Unmixed component signals (32-bit greyscale) from tissue sections stained with the lymphocyte and cytotoxic/NK panels [primary channel columns] with satisfactory **(A)** and non-satisfactory **(B)** binary mask (black-and-white) outputs [secondary thresholded channels columns] after running the dynamic image thresholding sequence.

Figure S2: Example colocalisation-based resolutions of cell detections with apparent double-positivity for two membranous markers

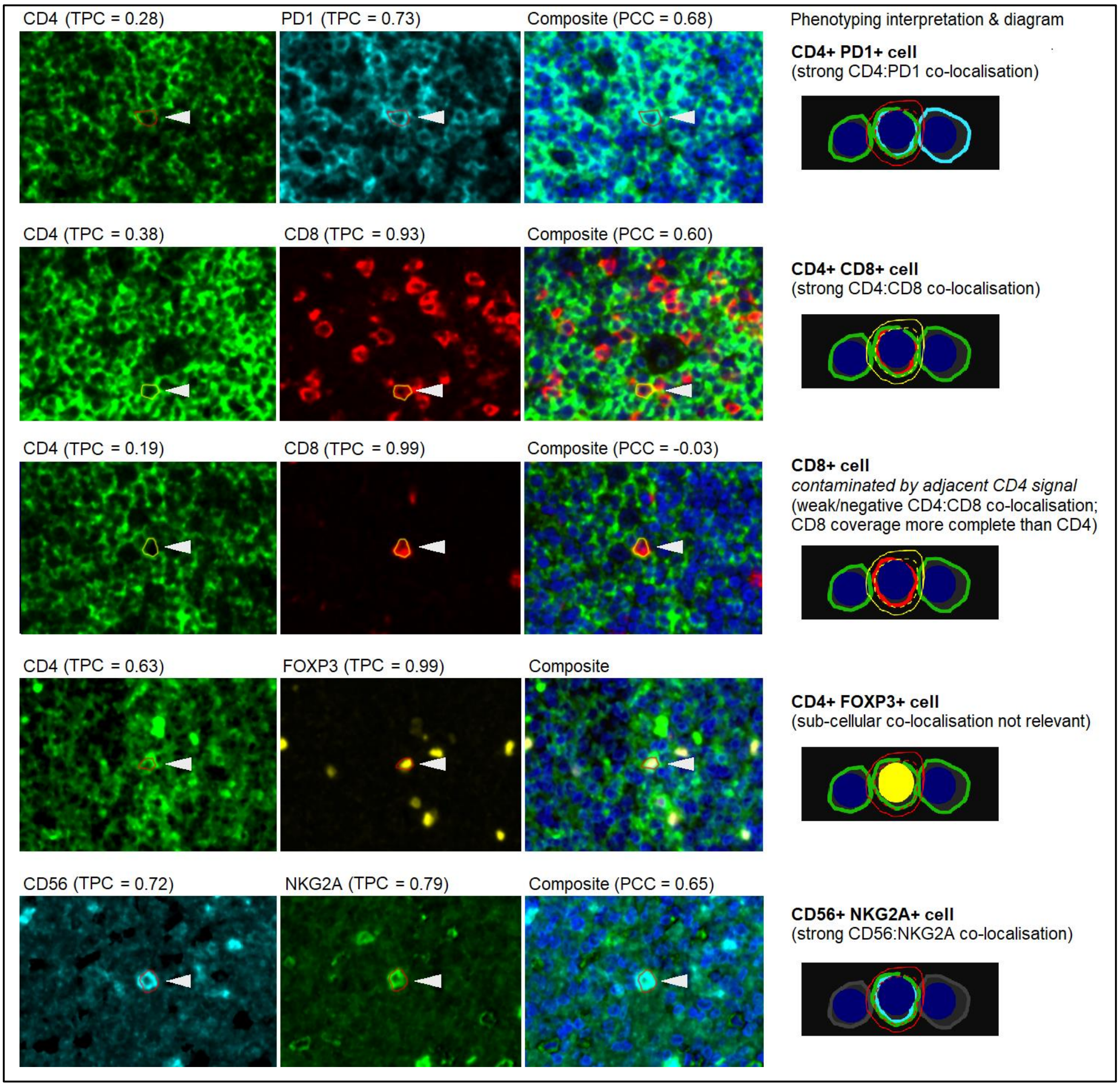

Legend: Example detection shown on each row: unmixed component signals from two markers with selected detection's thresholded positive coverage (TPC) measurement shown [1st and 2nd columns]; composite image of both markers with DAPI (blue) and Pearson Correlation Coefficient between signals shown [3rd column]; resolved decision with rationale and diagrammatic representation [final column].

Figure S3: Example evaluation of marker positivity determination

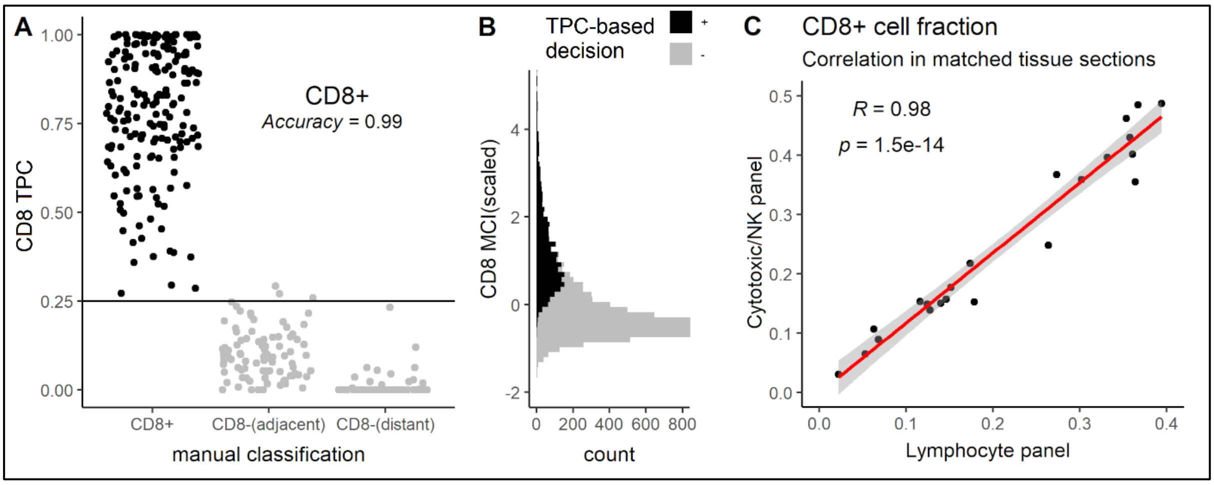

Legend: *TPC*= thresholded positive coverage; *MCI*= mean cell intensity; CD8 TPC measurements of evaluation subset detections (randomly selected but stratified to cover a full range of TPC values) grouped by human classified groundtruth, with subsequently selected threshold (horizontal line at 0.25) [A]; TPC-based decisions mapped to a histogram of MCI measurements, demonstrating effectiveness in extracting a sub-distribution of detections from the overall distribution [B]; Correlation between CD8+ cell fraction derived from matched lymphocyte and cytotoxic/NK panel-stained areas [C].

Figure S4: Example evaluation of co-localisation-based resolutions

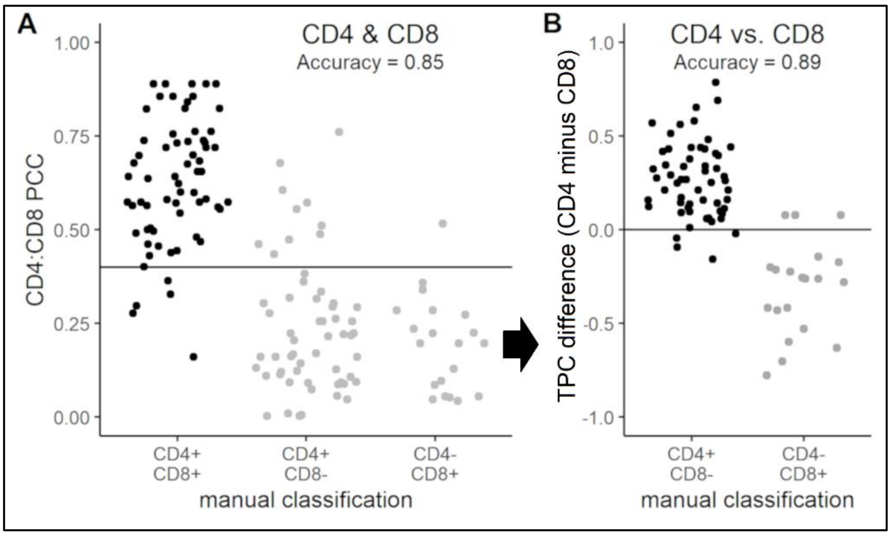

Legend: *CD4:CD8 PCC*= Pearson correlation coefficient between CD4 and CD8 primary channels; *TPC*= thresholded positive coverage; CD4:CD8 PCC measurements of evaluation subset detections (randomly selected but stratified to cover a full range of positive PCC values), with subsequently selected threshold (horizontal line at 0.4) [A]; and difference in TPC measurements of detections below PCC threshold [B]; both grouped by human classified groundtruth.

Figure S5: Additional lymphocyte panel phenotypes notable for their expansion in the single "Treg-high" THRLBCL case

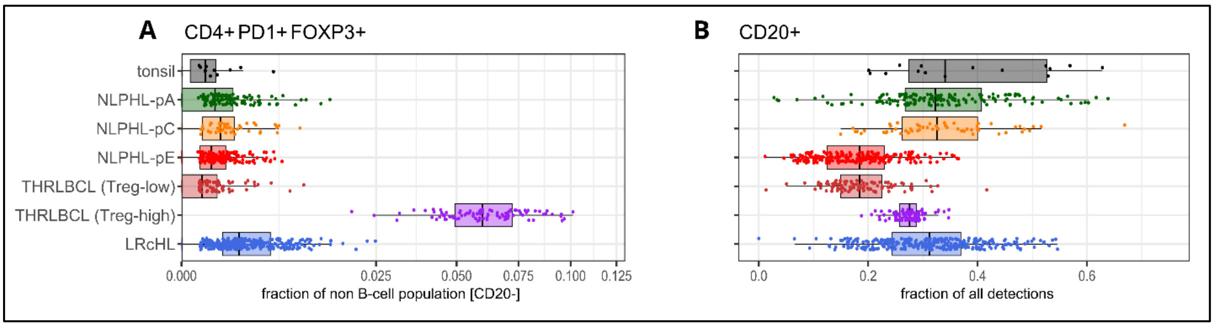

Legend: Fraction of CD4+PD1+FOXP3+ detections (possible T-follicular regulatory cells) out of the total CD20-negative non-B cell populations[A]; and fractions of CD20+ B-cells out of the total cell detection population [B]; both grouped by case type.

Figure S6: Additional macrophage panel phenotypes notable for their expansion in the single "Treg-high" THRLBCL case

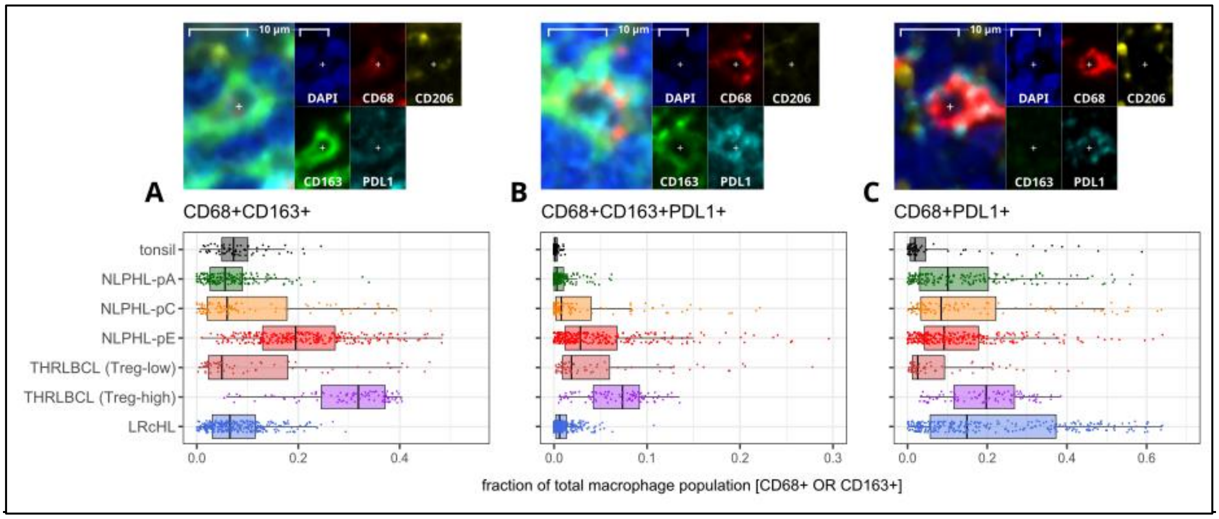

Legend: For each phenotype as indicated [A-C]: representative detection [upper: multispectral fluorescence composite image (left) and component channels (right) shown to scale as indicated); and phenotype fractions in image tiles quantified relative to the parent total macrophage population and grouped by case type [lower].
